# Awareness and Risk Reduction of Hypertension Among Adults in Ilala and Mkuranga Districts, Tanzania

**DOI:** 10.24248/eahrj.v9i1.834

**Published:** 2025-09-30

**Authors:** Alison Kabanda, Hadijah Ally Mbwana, Helena Aminiel Ngowi

**Affiliations:** a Africa Academy for Public Health, Dar-es-Salaam, Tanzania; b Department of Veterinary, Medicine and Public Health, Sokoine University of Agriculture, Morogoro, Tanzania; c Department of Human Nutrition and Consumer Sciences, Sokoine University of Agriculture, Morogoro, Tanzania.

## Abstract

**Background:**

Hypertension is one of the major global public health problems that has been associated with an increasing prevalence of cardiovascular diseases (CVDs) such as stroke and ischemic heart disease. This study aimed to assess awareness and the practice of risk reduction of hypertension among adults in Ilala and Mkuranga districts, Tanzania.

**Methods:**

A community based cross sectional study was conducted whereby a total of 295 participants were interviewed using a questionnaire adapted from the World Health Organization (WHO). Stepwise approach for chronic disease risk factor surveillance was used to obtain the socio-demographic information, knowledge, awareness, and attitudes regarding hypertension using a structured set of questions. Blood pressure was measured and recorded. Descriptive statistics were used to describe and summarize the study findings. Pearson Chi-square test was used to compare and determine the association between categorical variables and hypertension. Multiple logistic regression analysis was performed to determine predictors of hypertension.

**Results:**

Statistical association for all comparisons was set at *P*<.05. Hypertension prevalence was high (36.9%) with high proportion of hypertensive individuals being aged between 30 to 44 years (48.6%), married or cohabiting (70.6%), self-employed (59.6%), attained primary education level (59.6%), earn <TZS 250,000 per month (47.7) and living in rural Mkuranga (48.6%). Over a third of hypertensive individuals were unaware of their condition. Although over half of studied individuals had good knowledge of risk factors for hypertension, their risk reduction practices were limited. Only 44.4% engaged in physical activity, 7.5% quit smoking, and 9.2% reduced their alcohol intake. However, in multiple logistic regression age and knowledge maintained its significant association with hypertension. Tailored community interventions are urgently needed to improve awareness and enhance preventive practices against hypertension.

**Conclusion:**

This study underscores the urgent need for enhanced hypertension prevention strategies in study population. Significant gaps remain in awareness, risk perception, and adoption of preventive practices.

## BACKGROUND

Hypertension defined as SBP ≥140 mmHg or DBP ≥90 mmHg remains a major driver of cardiovascular morbidity and mortality and imposes substantial economic costs.^1,2^ Globally, adult hypertension has nearly doubled over three decades, with a disproportionate burden in Africa.^2^ In Tanzania, recent evidence suggests a prevalence around one-third of adults, with particularly low rates of awareness, treatment, and control.^3^

Across sub-Saharan Africa, awareness of hypertension is strikingly low, often below one in four adults, undermining early diagnosis and linkage to care.^4,5^ Low awareness is compounded by health-system constraints, sociocultural beliefs, and limited routine screening in primary care factors that collectively delay care-seeking and sustained control.^6^ Evidence from the region indicates that multi-pronged strategies community blood-pressure screening and referral, task-sharing with community health workers, integration of hypertension checks into routine primary care, mHealth reminders, salt-reduction education, and brief cessation counselling can improve awareness and risk reduction behaviors, especially when adapted to local contexts.^4^

This study focuses on Ilala (urban) and Mkuranga (rural) districts in coastal Tanzania to capture urban–rural contrasts that shape hypertension awareness and prevention. Ilala's denser health infrastructure and higher service availability differ markedly from Mkuranga's dispersed settlements, longer travel distances, and more limited facility access conditions that plausibly influence screening uptake, knowledge transfer, and feasibility of risk-reduction practices. Examining these settings side-by-side enables identification of context-specific barriers and intervention entry points that a single-site study could miss.

We are guided by a brief conceptual framework linking awareness, knowledge, attitudes, andpractices (AKAP): (1) Awareness (recognition of BP status and exposure to screening) enables (2) Knowledge (accurate understanding of risk factors and control measures), which shapes (3) Attitudes (perceived susceptibility, benefits, and self-efficacy) that ultimately drive (4) Risk-reduction practices (physical activity, diet/salt reduction, tobacco and alcohol cessation, weight control). Breakdowns at any step e.g., low awareness or misconceptions can weaken downstream behaviors and sustain high prevalence despite available services.

Accordingly, this community-based study assesses the prevalence of hypertension and examines awareness, knowledge, attitudes, and practice domains among adults in Ilala and Mkuranga. By quantifying awareness gaps and their correlates, and by comparing urban-rural patterns, the study aims to inform tailored, scalable interventions to raise awareness and strengthen prevention and control of hypertension in Tanzania.^3,4,7^

## METHODOLOGY

### Study Area

This study was conducted in Tanzania's Mkuranga and Ilala districts, which are located in the country's coastal and Dar es Salaam areas, respectively. While Mkuranga represents a rural setting with dispersed settlements, agricultural fields, limited access to healthcare and educational services, and the presence of traditional practices and cultural norms that may have an impact on health-related behaviours and/or outcomes, Ilala municipality represents the urban environment in terms of population density, economic activities, infrastructure development, and social service distribution, among other things.

### Research Design and Sampling Procedure

This study used a cross-sectional research design, with participants selected from within the population using a simple random sampling technique and the districts of Mkuranga and Ilala divided into discrete clusters using cluster sampling.

### Study Population and Criteria

The study involved sampling of adults 30 years and above living in Ilala and Mkuranga districts. This age criterion was strategically chosen because hypertension and related risk factors tend to manifest at this age.^7^ Pregnant women and those with mental and hearing disabilities were excluded from the study.

### Sample Size

The required sample size was calculated using Fisher's formula for estimating proportions:



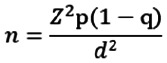



Whereby;

n = sample size, Z-Score = 1.96 (at 95% confidence level), P = known or estimated prevalence of hypertension 25.9%, d = margin of error (0.05).

The sample size was:



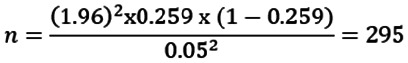



Whereas Mkuranga district = 72 and Ilala district 223, the larger proportion of participants from Ilala reflects its higher population density compared to Mkuranga, hence more clusters and households were selected from the urban district. While this reflects underlying demographics, it may overrepresent urban participants relative to rural populations.

### Data Collection and Pilot Testing

Data were collected using a structured questionnaire adapted from the WHO STEPwise approach for NCD surveillance^2^. Prior to main data collection, the tool was pilot-tested among 20 adults in a non-study district to ensure clarity, cultural appropriateness, and reliability. Necessary modifications were incorporated. Research assistants received one-week training in interviewing techniques, informed consent, and use of KoboCollect. Supervisors conducted daily spot-checks of completed forms to ensure accuracy and completeness. Double-entry validation was performed in SPSS to minimize errors.

Kobo toolbox was utilized for data input, and IBM-SPSS version 25 was used for analysis.

### Assessment of Awareness and Hypertension Risk Reduction Practices

Blood pressure was measured using a calibrated digital sphygmomanometer (Omron®) in accordance with WHO STEPS protocol. Each participant was seated and rested for at least 10 minutes before measurement. Three readings were taken at 5-minute intervals, and the average of the three readings was recorded. Appropriate cuff sizes were ensured, devices were calibrated daily, and all measurements were conducted by trained nurses who had completed a two-day standardized training. Hypertension was defined as SBP ≥140 mmHg and/or DBP ≥90 mmHg, or current use of antihypertensive medication. Awareness was defined as a positive response to having had blood pressure measured or being told by a health worker of having hypertension. Knowledge was assessed using seven items on risk factors, with scores ≥4 (≥50%) classified as “good knowledge,” consistent with prior studies (Linda, 2006; Mosha et al., 2017). Attitudes were measured using five Likert-scale items, with ≥3 favorable responses indicating a “positive attitude.” Risk-reduction practices were self-reported lifestyle modifications, including physical activity, reduced salt intake, alcohol and tobacco cessation, weight control, and dietary changes. Physical activity was categorized according to WHO guidelines, with light-intensity activities (walking, household chores, light gardening) distinguished from moderate-intensity activities (brisk walking, cycling, farming, or carrying moderate loads for at least 10 minutes continuously).

### Data Analysis

Descriptive statistics were used to describe and summarize the sociodemographic data using frequencies and percentages. Pearson Chi-square test was used to compare different categories and determine the association between categorical variables and hypertension. Logistic regression to show the relationship, predict the contribution of risk factors associated with hypertension, and compare the odds of exposure between being normal and hypertensive. Statistical association for all comparisons was set at *P*<.05.

### Ethical Considerations

A research permit was obtained from the Sokoine University of Agriculture (SUA). The participants were required to sign the consent form or apply a thumbprint (in ink), marking their consent to participate in the study after being given information on the study. Respondents were made aware of their freedom to not participate in the study as well as withdraw from the study, without negative consequences. Ethical clearance was obtained from the Tanzania National Institute for Medical Research (NIMR/HQ/R.8a/Vol.IX/4562).

## RESULTS

### Sociodemographic Characteristics of Study Participants

Table 1 presents the distribution of participants’ sociodemographic characteristics including age, sex, education, occupation, marital status, and household size, as well as the prevalence of hypertension within each category. This study involved 295 participants from both Ilala (75.6%) and Mkuranga (24.4%) districts, comprising females (50.8%) and males (49.2%). Most of the participants were 30 to 44 years of age (66.1%), and more than half (53.6%) had primary school education. The majority of participants (58.3%) were married, and (57.6%) were self-employed.

**TABLE 1:** Study Participants Sociodemographic Characteristics

	Examined (N=295)	Mkuranga (N = 72)	Ilala (N=223)
Sex	n (%)	n (%)	n (%)
Male	145 (49.2)	35 (48.6)	110 (49.3)
Female	150 (50.8)	37 (51.4)	113 (50.7)
Age			
30 – 44 years	195 (66.1)	48 (66.7)	147 (65.9)
45 – 64 years	80 (27.1)	20 (27.8)	60 (26.9)
>65 years	20 (6.8)	4 (6.9)	16 (6.7)
Marital status			
Single	72 (24.4)	18 (25.0)	54 (24.2)
Married/Cohabiting	196 (66.4)	48 (66.7)	148 (66.4)
Widowed/Divorced/Separated	23 (7.8)	6 (8.3)	17 (7.6)
Education			
No formal education	14 (4.7)	3 (4.2)	11 (4.9)
Primary school	158 (53.6)	39 (54.2)	119 (53.4)
Secondary school/college	114 (38.6)	28 (38.9)	86 (38.6)
University	9 (3.1)	2 (2.8)	7 (3.1)
Occupation			
Unemployed	50 (16.9)	12 (16.7)	38 (17.0)
Self-employed	204 (69.2)	50 (69.4)	154 (69.1)
Employed	41 (13.9)	10 (13.9)	31 (13.9)
Income per month (TZS)			
<250,000	151 (51.2)	37 (51.4)	114 (51.1)
250,000 – 500,000	92 (31.2)	22 (30.6)	70 (31.4)
>500,000	52 (17.6)	13 (18.1)	39 (17.5)

### Participants’ Knowledge, Attitude and Awareness of Own Hypertension Status and Associated Risk Factors

In the study population, most participants (66.1%) never examined blood pressure. The majority of hypertensive participants (32.2%) were unaware of their condition. Most participants (88.1%) had no family history of hypertension complications, and the majority of hypertensive (35.9%) never received treatment for hypertension. Most of the participants were aware that lifestyle influences risk factors for hypertension (88.1%), and diet control and exercise are essential in the management of hypertension (86.8%). The majority of participants were aware that regular physical exercise is essential to prevent hypertension (71.9%), Obese/overweight is a risk factor for hypertension (67.1%), Alcohol consumption causes hypertension (64.4%), and Smoking tobacco causes hypertension (57.6%). The majority of the participants disagreed that the cost of preventing hypertension is high (61.7%), minerals are essential in controlling hypertension and its complications (66.4%), and that there are some traditional medicines for preventing risk factors of hypertension (60.7%). However, more than half of the participants agreed that excessive salt intake is harmful to health (59.7%), and lifestyle modification (62.4%) is useful in controlling high blood pressure (Table 2).

**TABLE 2:** Participants’ Knowledge, Attitude and Awareness of Own Hypertension Status and Associated Risk Factors

	All (%)	Mkuranga (%)	Ilala (%)
Never measured blood pressure	66.4	66.7	66.5
Never been told to have high blood pressure	90.2	90.3	90.1
Not told to have high blood pressure in last 12 months	95.9	95.8	95.9
No family history of high blood pressure	88.1	87.5	88.3
Aware that lifestyle influences risk factors for hypertension	88.1	87.5	88.3
Aware that smoking tobacco causes hypertension	57.6	56.9	57.8
Aware that alcohol consumption causes hypertension	64.4	63.9	64.7
Aware that being obese or overweight is a risk for hypertension	67.1	66.7	67.3
Aware that diet control and exercise are essential in the management of hypertension	86.8	86.1	87.0
Aware that regular exercise is essential for prevention of hypertension	71.9	72.2	71.7
Agree that the cost of preventing hypertension is high	8.3	38.9	38.1
Agree that excessive salt intake is harmful to health	59.7	59.7	59.6
Agree that minerals are essential in controlling hypertension and its complications	33.6	33.3	33.6
Agree that lifestyle modification is useful in controlling high blood pressure	62.4	62.5	62.3
Agree that risk factors for hypertension can be prevented without taking medication	45.8	45.8	45.7
Agree that there are some traditional medicines for preventing risks of hypertension	39.3	38.9	39.5

### Risk Reduction Practices of Hypertensive Study Participants

About risk reduction practices on hypertension among hypertensive study participants. 33% of them did exercise more. Most of the participants (79.8%) reported to reduce salt intake and improve their diets (555%). Few participants (10.1%) had reduced alcohol consumption (7.3%) quit smoking tobacco products. Less than half participants exercised more (42.7%) and controlled weight (35.3%) Table 3.

**TABLE 3:** Risk Reduction Practices of Hypertensive Study Participants

	All (N=109) n (%)	Mkuranga (N=35) n (%)	Ilala (N=74) n (%)
Exercise more	36 (33)	12 (34.3)	24 (32.4)
Have reduced salt intake	87 (79.8)	28 (80)	59 (79.7)
Have reduced alcohol consumption	11 (10.1)	4 (11.4)	7 (9.5)
Stopped smoking	8 (7.3)	3 (8.6)	5 (6.8)
Improved diet intake	60 (55)	19 (54.3)	41 (55.4)

### Prevalence of Hypertension and Socio-demographics

Among the 109 hypertensive participants, slightly more than half were male (55.0%). Nearly half (48.6%) were aged 30 to 44 years, followed by 39.4% aged 45–64 years, and 11.9% aged ≥65 years. The majority were married or cohabiting (70.6%), had attained primary education (59.6%), and were self-employed (59.6%). Regarding income, almost half (47.7%) earned <250,000 TZS monthly. These patterns were consistent across both districts, with Mkuranga and Ilala showing broadly similar distributions (Table 4).

**TABLE 4:** Prevalence of Hypertension and Socio-demographics

	All (N=109)	Mkuranga (N=35)	Ilala (N=74)
Sex	n (%)	n (%)	n (%)
Male	60 (55.0)	19 (54.3)	41 (55.4)
Female	49 (45.0)	16 (45.7)	33 (44.6)
Age			
30 – 44 years	53 (48.6)	17 (48.6)	36 (48.6)
45 – 64 years	43 (39.4)	14 (40.0)	29 (39.2)
>65 years	13 (11.9)	4 (11.4)	9 (12.2)
Marital status			
Single	20 (18.3)	6 (17.1)	14 (18.9)
Married/Cohabiting	77 (70.6)	25 (71.4)	52 (70.3)
Widowed/Divorced/Separated	12 (11.0)	4 (11.4)	8 (10.8)
Education			
No formal education	8 (7.3)	3 (8.6)	5 (6.8)
Primary school	65 (59.6)	21 (60.0)	44 (59.5)
Secondary school/college	32 (29.4)	10 (28.6)	22 (29.7)
University	4 (3.7)	1 (2.9)	3 (4.1)
Occupation			
Unemployed	25 (22.9)	8 (22.9)	17 (23.0)
Self-employed	65 (59.6)	21 (60.0)	44 (59.5)
Employed	19 (17.4)	6 (17.1)	13 (17.6)
Income per month (TZS)			
<250,000	52 (47.7)	17 (48.6)	35 (47.3)
250,000 – 500,000	37 (33.9)	12 (34.3)	25 (33.8)
>500,000	20 (18.3)	6 (17.1)	14 (18.9)

### Predictors of Hypertension

The relationship between risk factors and hypertension remained significant for age (*P*<.001), family history of hypertension (*P*=.031), current smoking (*P*=.001), and current alcohol use (*P*<.001) in the multivariable model. Other factors including education, occupation, income, diet, and salt reduction were not significantly associated with hypertension (*P*>.05) (Table 5).

**TABLE 5:** Predictors of Hypertension

Characteristics	Unadjusted		Adjusted	
	OR	95% CI	OR	95% CI
Sex				
Male	Ref		Ref	
Female	1.05	0.65–1.70	1.02	0.58–1.78
Age				
30 – 44 years	0.15	0.05–0.40	0.05	0.01–0.19
45 – 64 years	0.30	0.12–0.72	0.13	0.03–0.56
>65 years	Ref		Ref	
Marital status				
Single	0.90	0.40–2.00	0.95	0.38–2.40
Married/Cohabiting	1.10	0.55–2.30	1.08	0.50–2.35
Widowed/Divorced/Separated	Ref		Ref	
Education				
No formal education	1.20	0.35–4.10	1.10	0.30–4.00
Primary school	1.05	0.40–2.70	1.00	0.35–2.85
Secondary school/college	0.95	0.35–2.60	0.92	0.33–2.55
University	Ref		Ref	
Occupation				
Unemployed	1.15	0.52–2.55	1.10	0.48–2.52
Self-employed	1.05	0.60–1.90	1.03	0.55–1.95
Employed	Ref		Ref	
Income per month (TZS)				
<250,000	1.20	0.62–2.40	1.15	0.58–2.30
250,000 – 500,000	1.05	0.50–2.10	1.00	0.48–2.05
>500,000	Ref		Ref	
Knowledgeable				
Yes	Ref		Ref	
No	0.30	0.12–0.70	0.24	0.103–0.55
Awareness				
Yes	Ref		Ref	
No	0.95	0.55–1.65	0.92	0.50–1.68
Attitude				
Positive	Ref		Ref	
Negative	0.85	0.42–1.70	0.82	0.40–1.75
Risk reduction practice				
Good	Ref		Ref	
Poor	2.80	1.20–6.50	3.29	1.11–9.76

## DISCUSSION

This study investigated the prevalence of hypertension alongside levels of awareness, attitudes, and risk-reduction behaviours among adults in Ilala and Mkuranga districts. The study group was largely composed of individuals with primary education, females, younger adults aged 30 to 44 years, married participants, and residents from the urban setting of Ilala.

The findings point to a considerably high prevalence of hypertension across both districts, with rural Mkuranga reporting notably higher rates than urban Ilala. While this pattern could partly be explained by the smaller rural sample size, it also likely reflects contextual realities. Elevated rural prevalence may be linked to structural barriers such as limited availability of regular health services, scarcity of screening programs, long distances to healthcare facilities, and persistent reliance on traditional healing practices instead of biomedical care. Conversely, those living in urban Ilala may have benefited from closer proximity to hospitals, more consistent access to health information, and more frequent incidental screening opportunities, although their lifestyles characterized by physical inactivity and dietary shifts still predispose them to hypertension.

The urban–rural disparity in access to health services appears central to explaining these outcomes. Reports from Mkuranga indicate that some participants travel up to three hours or incur significant transport costs to reach a health facility.^3^ These barriers discourage preventive health-seeking, delay diagnosis, and increase the likelihood of uncontrolled hypertension. The evidence highlights the necessity of decentralizing hypertension-related services, strengthening the role of community health workers, and embedding blood pressure checks into existing rural outreach and primary healthcare initiatives.

A striking paradox also emerged: while more than half of the respondents exhibited “good knowledge” of hypertension risk factors, over one-third of hypertensive individuals were unaware of their own condition. This distinction underscores the gap between theoretical understanding and personal health recognition. People may know that alcohol, tobacco use, and high salt intake are risk factors, but without regular measurement of blood pressure, they fail to translate this knowledge into awareness of their own status. Similar discrepancies have been observed in neighboring countries such as Kenya and Uganda, where knowledge levels exceeded 60% but personal awareness was below 25%.^5,8^ Such gaps underline the urgency of transforming knowledge into actionable behavior through systematic screening, individualized counseling, and structured follow-up services.

Regionally, the prevalence observed in this study aligns with figures reported from Uganda (31–34%) and Kenya (29–36%) but surpasses those found in Rwanda (19–24%).^9^ Awareness levels in Tanzania, with 32.2% of hypertensive individuals unaware of their condition, mirror those in Kenya (27%) yet remain lower compared to South Africa (46%), emphasizing the shared African challenge of underdiagnosis and late detection.

The implications for health policy are significant. Although Tanzania's NCD Strategic Plan underscores the importance of early detection, the actual roll-out remains inadequate, particularly in underserved rural areas.^10^ Policy responses must therefore prioritize strengthening primary healthcare systems through integration of hypertension screening into maternal and child health services, HIV programs, and other established care platforms, ensuring maximum reach at minimal cost. In parallel, rural health workers should be trained and equipped with affordable diagnostic tools to facilitate timely diagnosis and management.

Future interventions must extend beyond conventional awareness campaigns. Recommended approaches include: (1) locally organized screening drives in community hubs such as markets, churches, and workplaces; (2) provision of subsidized mobile clinics to serve hard-to-reach areas; (3) adoption of SMS and mobile health applications to remind individuals about regular screening and lifestyle adjustments; (4) establishment of peer-support systems to aid in smoking and alcohol cessation; and (5) workplace wellness programs to promote physical activity, especially in urban populations.

Although the study participants showed fair knowledge of hypertension risk factors, limited personal awareness and weak engagement in risk-reduction strategies reveal persistent shortcomings in prevention. Rural communities remain disproportionately vulnerable due to service accessibility barriers. Tailored, context-sensitive programs and evidence-based policies are urgently needed to close the gap between awareness and action, thereby reducing the rising burden of hypertension across Tanzania and the broader East African region.

## CONCLUSION

This study underscores the urgent need for enhanced hypertension prevention strategies in study population. Significant gaps remain in awareness, risk perception, and adoption of preventive practices. There is a clear need for community-based screening, targeted health education, and behavior change interventions tailored to local contexts. Efforts should focus on dispelling misconceptions such as how people think that hypertension is a disease for rich people only, promoting affordable lifestyle modifications, and improving access to preventive and treatment services. Policymakers and health stakeholders must prioritize culturally appropriate strategies to reduce the burden of hypertension and its complications in these communities.
